# Hydroethanolic Extract of* Strychnos pseudoquina* Accelerates Skin Wound Healing by Modulating the Oxidative Status and Microstructural Reorganization of Scar Tissue in Experimental Type I Diabetes

**DOI:** 10.1155/2017/9538351

**Published:** 2017-09-13

**Authors:** Mariáurea M. Sarandy, Rômulo D. Novaes, Antônio A. Xavier, Camilo E. Vital, João P. V. Leite, Fabiana C. S. A. Melo, Reggiani V. Gonçalves

**Affiliations:** ^1^Department of General Biology, Federal University of Viçosa, 35570-000 Viçosa, MG, Brazil; ^2^Institute of Biomedical Sciences, Department of Structural Biology, Federal University of Alfenas, 37130-001 Alfenas, MG, Brazil; ^3^Department of Biochemistry and Molecular Biology, Federal University of Viçosa, 35570-000 Viçosa, MG, Brazil; ^4^Center of Biomolecules Analysis (NuBioMol), Federal University of Viçosa, 35570-000 Viçosa, MG, Brazil; ^5^Department of Animal Biology, Federal University of Viçosa, 35570-000 Viçosa, MG, Brazil

## Abstract

The effect of topical application of ointment based on* Strychnos pseudoquina* hydroethanolic extract in the cutaneous wounds healing in diabetic rats was evaluated. Samples of* S. pseudoquina* were submitted to phytochemical prospection and* in vitro* antioxidant assay. Thirty Wistar rats were divided into 5 groups: Sal-wounds treated with 0.9% saline solution; VH-wounds treated with 0.6 g of lanolin cream (vehicle); SS-wounds treated with silver sulfadiazine cream (10 mg/g); ES5- and ES10-wounds treated with an ointment of* S. pseudoquina* extract, 5% and 10%, respectively. Fragments of wounds were removed for histological and biochemical analysis every 7 days during 21 days. ES showed equivalent levels per gram of extract of total phenols and flavonoids equal to 122.04 mg for TAE and 0.60 mg for RE. The chlorogenic acid was one of the major constituents.* S. pseudoquina* extract presented high antioxidant potential* in vitro*. ES5 and ES10 showed higher wound healing rate and higher amount of cells, blood vessels, and type III and I collagen. The oxidative stress markers were lower in the ES5 and ES10 groups, while the antioxidants enzymes levels were higher. Ointment based on* S. pseudoquina* extract promotes a fast and efficient cutaneous repair in diabetic rats.

## 1. Introduction

Wound healing is a complex and organized process characterized by tissue changes, which include increased vascularization, cell proliferation, and extracellular matrix synthesis [1, 2]. These mechanisms not only accelerate wound healing, but also are responsible for restoring injured tissue function [3]. The cutaneous repair process can be divided into four complementary phases. The first phase is known as hemostasis [4], the second as inflammatory [5, 6], the third as proliferative, and the fourth as remodeling [3, 7]. The cells recruited in the inflammatory phase are responsible for cytokines and growth factors release that will mediate the processes of migration, proliferation, and cell differentiation that are typical of the proliferative phase [8, 9].

Among cytokines and growth factors released during the inflammatory phase we can highlight proinflammatory mediators such as interleukin-1 (IL-1), TNF-*α*, and IFN-*γ*, which stimulate the diapedesis and cell proliferation [10]. Some mediators, like TGF-*β*, are preformed in the organism [11, 12] and produced by different cells, which are responsible for cellular chemotaxis and the production of vessels and fibers, accelerating the tissue remodeling process [13, 14]. The tissue events stimulated by TGF-*β* provide proper nutrition and energy for cells to synthesize a new extracellular matrix rich in blood vessels and type III collagen, which will provide the scaffold for type I collagen synthesis [15, 16].

The formation of reactive oxygen species (ROS) occurs during the repair process, which might lead to a mechanism known as tissue oxidative stress that happens mainly in the inflammatory phase, along with the migration of phagocytes responsible for respiratory explosion [17, 18]. To protect the organism from this oxidative damage antioxidant enzymes are produced, such as superoxide dismutase (SOD), which converts superoxide radical (O_2_^∙−^) into hydrogen peroxide (H_2_O_2_) [19–21] and catalase (CAT) that converts H_2_O_2_ to H_2_O and O_2_ [22, 23].

Diabetes is a pathology associated with a severe impairment in cutaneous repair, mainly due to vascular and cellular alterations, which reduce the supply of oxygen and nutrients to the cells, resulting in insufficient and abnormal deposition of collagen fibers followed by a delay in the wound healing process [10, 24, 25]. The use of medicinal plants for treating cutaneous wounds in diabetes has been increasing, since the components found on the extracts of these plants usually act in different metabolic pathways involved in the repair process [24, 26]. Flavonoids, other polyphenols, and alkaloids are known for accelerating the healing process due to its anti-inflammatory, antimicrobial [27, 28], antioxidant [29, 30], and immune stimulating properties [31].

*Strychnos* has more than 200 species distributed around tropical regions of the world [32]. In regions of Brazilian Cerrado, the species* Strychnos pseudoquina* A. St. Hil (Loganiaceae family) also known as “Quina do Cerrado” is mainly indicated for malaria treatment [33] and for gastrointestinal lesions [34, 35]. Phytochemical studies carried out with* S. pseudoquina*'s stem bark collected at Minas Gerais, a Brazilian state, has demonstrated the majority presence of two flavonoids: quercetin 3-*O*-methyl ether and strychnobiflavone [36]. This last biflavonoid showed antileishmanial [37] and anti-inflammatory activities [38].

Emphasizing the importance of understanding the therapeutic effects of this plant, this study aims to evaluate the effects of* S. pseudoquina* extract in different concentrations on morphological and biochemical characteristics during the repair of second intention cutaneous wounds in diabetes.

## 2. Material and Methods

### 2.1. Sample Collection and Extract Preparing

All protocols developed in this study were carried out according Souza [39]. Stem bark samples of* S. pseudoquina* were collected in Rio Verde, Goiás State, Brazil, in September 2014 (latitude −17°47′53′′; longitude −50°55′41′′) and deposited in the EPAMIG-BH herbarium (registration PAMG 57079). Dried and powdered samples (1.000 g) were subjected to selective sequential extraction by percolation using solvents of increasing polarity, namely, n-hexane, ethyl acetate, and ethanol/water (9 : 1, v/v). The latter extraction cycles, after the removal of less polar compounds, were concentrated using a rotatory evaporator and then lyophilized yielding a hydroethanolic extract denominated ES. The yield was 13.2% (w/w).

### 2.2. Phytochemical Prospection

The presence of secondary metabolites, such as flavonoids, tannins, coumarins, anthraquinones, triterpenes/steroids, saponins, and alkaloids, was investigated by TLC analysis using silica gel chromatography GF_254_ (Sigma-Aldrich®). Different systems of mobile phases and specific visualization reagents were used according to procedures described by Harbone [40]. The results obtained by chromatography were compared with respective standards.

### 2.3. Determination of Phenolic Compounds

Total polyphenols content of the ES was examined by Folin-Ciocalteu reagent using tannic acid as the standard, and the absorbance was measured at 760 nm following a previously reported method [41] with slight modifications. Folin-Ciocalteu reagent (0.5 mL), 1 mL of aqueous Na_2_CO_3_ (7.5%), and distilled water (8.3 mL) were added to a test tube, followed by the addition of 200 *μ*L of ES solution (initially 5.0 mg/mL resuspended in ethanol). The total tannin was expressed as mg of tannic acid equivalent (TAE)/g of ES.

The total flavonoids were quantified by the aluminum chloride method using rutin as the reference compound and the absorbance measured in a UV-Visible spectrometer after 15 min of reaction at room temperature in the dark [41, 42]. An aliquot of 10.0 mg of the ES was suspended by sonication in 1.0 mL of methanol and then 8 mL of methanol, 0.6 mL of acetic acid, and 2.5 mL of 8% aluminum chloride hexahydrate in methanol (w/v) were added. The total volume of 25 mL was completed with methanol. After 20 minutes, the absorption was read at 420 nm. The flavonoid content in the extract was expressed as mg of rutin equivalent (RE) per g of dry extract. All of the tests were conducted in triplicate.

### 2.4. UPLC-MS/MS Instrumentation and Analytical Conditions

The sample was automatically injected (5 *μ*l) in the system LC-MS/MS using an Agilent 1200 Infinity Series coupled to a mass spectrometry type triple Quadrupole (QqQ), model 6430 Agilent Technologies. Chromatographic separation was carried out on a column Zorbax Eclipe Plus C18 (1.8 *μ*m, 2.1 × 50 mm) (Agilent) in series with a guard column Zorbax SB-C18, 1.8 *μ*m (Agilent). The solvent used was (A) acetic acid 0.02% in water and (B) acetic acid 0.02% in acetonitrile in a gradient of time/% B: 0/5; 11/60; 13/95; 17/95; 19/5; 20/5. The solvent flow rate was 0.3 ml/min in a column temperature of 23°C. The ionization method used in the mass spectrometry was ESI (Electrospray Ionization) following these conditions: gas temperature of 300°C, nitrogen flow rate of 10 L/min, nebulizer pressure of 35 psi, and capillary voltage of 4000 V [41, 42].

### 2.5. Determination of Phenolic Compounds in the ES Extract by UPLC-ESI-MS/MS

The equipment was operated on MRM (Multiple Reaction Monitoring) mode. The mass of the precursor ion/fragment established was monitored by fragmentation tests of each molecule. For the identification of the compounds, 26 standard phenolic compounds (benzoic acid, caffeic acid, ferulic acid, isoferulic acid, chlorogenic acid, neochlorogenic acid, cinnamic acid, p-coumaric acid, syringic acid, sinapic acid, sinapyl alcohol, trans-cinnamic acid, 4-hydroxybenzoic acid, 3,5-dihydroxy benzoic acid, 4-hydroxy-3-methoxycinnamic acid, 4-hydroxyflavone, 7-hydroxyflavone, quercetin, apigenin, daidzein, coumarin, genistein, naringenin, catechin, vanillin, and curcumin) were analyzed using peaks with *m*/*z* relative by individual standards. Besides these, the presence of the two flavonoids already isolated from* S. pseudoquina*, quercetin 3-O-methyl ether, and strychnobiflavone were also investigated. A calibration curve (1 a 500 ng/mL) using the standard of all compounds was generated to determine the absolute quantification. The generated data were analyzed in the software “MassHunter Workstation” to obtain the peak areas for the samples, and the result was expressed in mg/g of ES extract.

For the sample preparation, 5.0 mg of ES was suspended with 1 mL methanol (100%), sonicated for 10 min at 25°C in an ultrasonic water bath, and successively diluted to a concentration of 10 *μ*g/mL. Finally, the extract was filtered through a 0.22 m syringe-membrane filter and aliquots of 5 *μ*L were injected into the UPLC-MS/MS system under the optimized analytical conditions. The quantitative result was expressed in micrograms per g (mg/g of ES).

### 2.6. *In Vitro* Antioxidant Potential

The antioxidant activity of* S. pseudoquina* extract was determined by the stable organic free radical DPPH (2,2-diphenyl-1-picrylhydrazyl) method. The DPPH method is based on reducing DPPH by antioxidants, which reduces the absorbance at 515 nm. Solutions were prepared from* S. pseudoquina* extract and hydroxytoluene butyl (antioxidant standard) in different concentrations (1 to 100 *μ*g/mL). An aliquot of each solution (0.3 mL) was added to a 2.7 mL DPPH solution (0.07 mM) and stood for 30 min in the dark. The absorbances were obtained by spectrometry at 515 nm. The DPPH radical scavenging activities were calculated according to the following equation: % DPPH radical scavenging = [(*A*_control_ − *A*_sample_)/*A*_control_] × 100.

### 2.7. Formulation Preparation

The lyophilized dry extract at concentrations of 5% (ES5) and 10% (ES10) was emulsified in lanolin. Silver sulfadiazine cream (SS) (1%) (Rexin Pharmaceuticals Pvt. Ltd.) was used as positive control [42, 43].

### 2.8. Animals

Thirty male Wistar rats* (Rattus norvegicus)* (198.25 ± 26.11 g) of 5 weeks of age were obtained from Federal University of Viçosa, Minas Gerais State, Brazil. Food and water were provided ad libitum. All the animals were kept in individual cages that were cleaned daily and under controlled conditions of temperature (22 ± 2°C), humidity (60–70%), and a 12-hour light/dark cycle. All the experiments were approved by the Animal Ethics Committee of the Federal University of Viçosa (registration n° 730/2014).

### 2.9. Diabetes Mellitus Induction and Experimental Design

**Figure 1:**
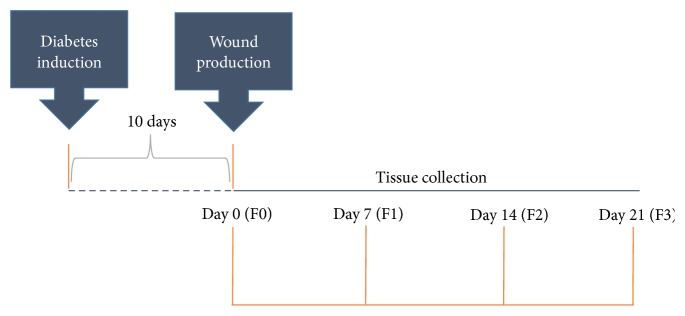
Experimental design: diabetes induction, 10 days later, wounds opening, and monitoring of the healing process for 21 days. At every 7 days, tissue was collected from the different wounds. Intact tissue (F0), healing tissue with 7 days (F1), healing tissue with 14 days (F2), and healing tissue with 21 days (F3).

Diabetes was induced by a single injection of streptozotocin (60 mg/kg) (Sigma, St. Louis, USA). Ten days after induction, blood glucose levels were monitored and animals with levels above 220 mg/dl were considered diabetics. After the confirmation of diabetes the animals were anesthetized by intramuscular injection of ketamine (50 mg/kg) and xylazine (20 mg/kg). Three circular wounds of 12 mm in diameter were made through surgical incision in the skin and subcutaneous tissue until the dorsal muscular fascia was exposed [44–46]. The wounds were located on the animal's back and organized in the cranial, middle, and caudal regions. The animals were separated in five groups with six animals in each group: (1) Sal (control): diabetic rats treated with 0.9% saline solution; (2) VH (vehicle): diabetic rats treated with 0.6 g of lanolin cream; (3) SS (silver sulfadiazine-positive control): diabetic rats treated with silver sulfadiazine (10 mg/g); (4) ES5: diabetic rats treated with an ointment of 5%* S. pseudoquina* extract; and (5) ES10: diabetic rats treated with an ointment of 10%* S. pseudoquina* extract. The wounds were daily cleaned with 0.9% saline solution before each treatment, which lasted for 21 days. After this period, the animals were euthanized by cardiac puncture. Experimental design is shown in Figure 1.

### 2.10. Wound Area and Contraction Index

The closure of the wounds was evaluated every 7 days in digitalized images with the dimensions of 320 × 240 pixels (24 bits/pixel) obtained using a digital camera (W320 Sony, Tokyo, Japan) [45, 46]. The wound area was calculated by computerized planimetry using the software Image-Pro Plus 4.5 (Media Cybernetics, Silver Spring, USA). The wound contraction index (WCI) was calculated using the ratio (Ao − AI)/Ao × 100, where Ao is the initial wound area (day 0) and AI is the wound area measured at the end of the experiment (day 21). For histological and biochemical analysis, scar tissue was collected from cranial (F1), middle (F2), and caudal (F3) wounds on days 7, 14, and 21, respectively [46].

### 2.11. Histological Analysis

Tissue fragments were fixed in Karnovsky's solution, dehydrated in ethanol, cleared in xylol, and embedded in paraffin [47, 48]. Semiserial 4 *μ*m thick histological sections were obtained using a rotary microtome using 1 in every 20 sections to avoid repeating analysis of the same histological area. The sections were stained with toluidine blue for analysis of the mast cells [47, 48] and, in order to determine tissue cellularity, 10 histological fields (×40 objective lens, CX40® light microscope, Olympus, Tokyo, Japan) were randomly sampled and analyzed, with total area of 1.53 × 10^6^ *μ*m^2^ [49, 50].

The sections were stained with hematoxylin and eosin (HE) for analysis of the fibroblasts and blood vessels. Sirius Red (Sirius Red F3B, Mobay Chemical Co., Union, NJ, USA) stain was used to differentiate collagen fibers under polarizing microscopy (Sigma, St. Louis, Mo, USA) [51, 52], and resorcin-fuchsin was used to differentiate elastic fibers [46]. The slides were visualized using a BX-60® light microscope (Olympus, Tokyo, Japan) connected to a digital camera (QColor 3®, Olympus). A total area of 1.53 × 10^6^ *μ*m^2^ was submitted to stereological analysis. Ten histological fields were randomly sampled in each skin section using a ×20 objective lens. A grid containing 300 points was superimposed over each image. The stereological parameters of volumetric density (Vv) were calculated by counting the points that occurred over fibroblasts, blood vessels, type III and type I collagen fibers, and elastic fibers using the ratio: Vv = (PP/PT) × 100, where PP is the number of points occurring over the structure of interest and PT is the total number of grid points [45, 46]. Collagen fibers were analyzed according to the differential properties of birefringence, since thick collagen fibers (type I) appear in shades of bright colors ranging from red to yellow whereas thin reticular fibers (collagen type III) appear bright green under polarization [45, 52].

### 2.12. TGF-*β* Expression Analysis

Scar tissue samples collected on days 7 (F1) and 14 (F2) were frozen at −80°C and homogenized in PBS, pH 7.4 buffer containing 0.05% of Tween, and centrifuged at 3.500*g* for 30 minutes. TGF-*β* levels on the supernatant were analyzed using immunoassay kits by ELISA method (Boster Biological Technology Ltd., China) following the fabricant's protocol.

### 2.13. Oxidative Stress Analysis

Tissue fragments were collected from each wound, rapidly frozen in liquid nitrogen, and stored in a freezer at −80°C. The samples were homogenized in phosphate buffered saline (PBS) and centrifuged at 5°C [45, 46]. The supernatant was used for the analysis of carbonyl proteins using the protocol described by Jana et al. [53]. Malondialdehyde (MDA) was measured according to the protocol reported by Gutteridge and Halliwell [54]. Catalase (CAT) activity was evaluated by measuring the decomposition rate of hydrogen peroxide (H_2_O_2_) [55] and superoxide dismutase (SOD), according to the Siddiqui et al. [56] protocol. The biochemical data were normalized in relation to total protein levels in the supernatant.

### 2.14. Statistical Analysis

Results are expressed as mean and standard deviation (mean ± SD). Data distribution was evaluated by the D'Agostino-Pearson test. The parametric distribution data was compared using one-way analysis of variance (ANOVA) followed by Newman-Keuls test. For nonparametric distributed data, a Kruskal-Wallis test was performed. Statistical significance was set at *p* < 0.05.

## 3. Results

### 3.1. Phytochemical Prospection and Quantification of Phenolic Compounds

The phytochemical prospection of* S. pseudoquina* extract indicated tannins, flavonoids, and alkaloids. The UHPLC-ES-MS/MS led to the identification of chlorogenic acid as one of the major constituents based on their molecular formula, fragmentation pattern, and comparison with the retention times of standards commercially available. In this method, the sample was compared qualitatively with standards of 28 phenolic compounds, including the flavonoids quercetin 3-O-methyl ether and strychnobiflavone, previously identified in the stem bark of* S. pseudoquina*. Quantification of the total phenolic compounds in ES determined the content of total polyphenolic compounds (63.18 mg TAE/g of ES) and flavonoids (0.60 mg RE/g of ES).

### 3.2. *In Vitro* Antioxidant Potential

The antioxidant potential, which corresponds to the extract concentration required to scavenge 50% of the initial concentration of DPPH radical (EC_50_), was of 80.09 *μ*g/mL, whereas for the BHT solution EC_50_ was of 5.51 *μ*g/mL.

### 3.3. Wound Area and Wound Contraction Index

**Figure 2:**
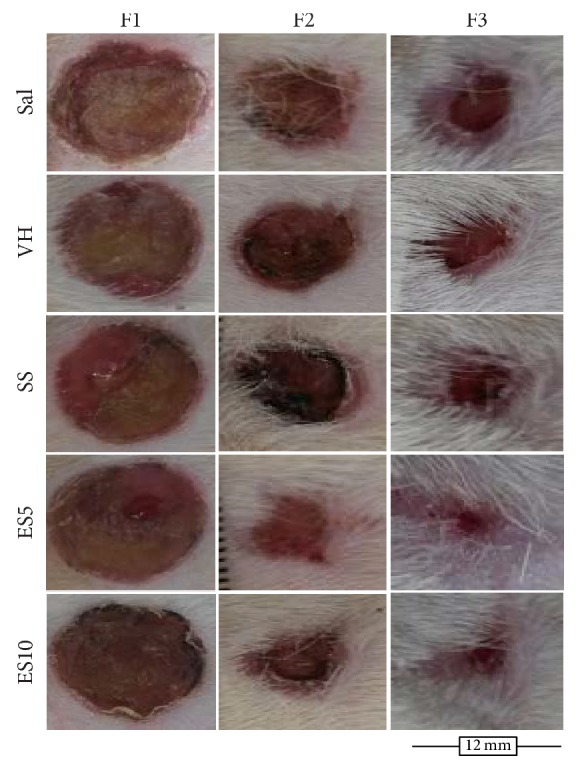
Second intention wound healing showing the evolution of cutaneous repair in diabetic rats treated with an ointment of* Strychnos pseudoquina* extract. Sal: 0.9% saline solution; VH: vehicle (lanolin cream); SS: silver sulfadiazine cream (10 mg/g); ES5 and ES10:* S. pseudoquina* 5% and 10%, respectively. F1, F2, and F3 = scar tissue on days 7, 14, and 21, respectively.

The wound area was smaller on days 7, 14, and 21 in groups ES5 and ES10 when compared to Sal and VH. These results are shown on Figure 2. There was no difference in the wound contraction index in the analyzed groups (Table 1).

### 3.4. Histopathological Results

**Figure 3:**
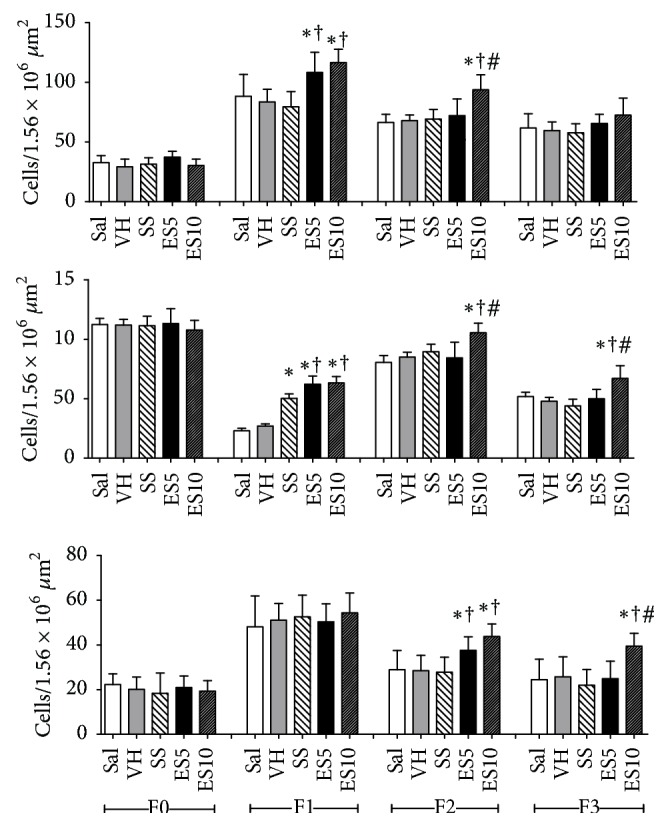
Total number of cells, mastocytes, and blood vessels in cutaneous wounds of diabetic rats treated with an ointment of* Strychnos pseudoquina* extract. Sal: 0.9% saline solution; VH: vehicle (lanolin cream); SS: silver sulfadiazine (cream 10 mg/g); ES5 and ES10:* S. pseudoquina* 5% and 10%, respectively. F0 = intact tissue on day 0; F1, F2, and F3 = scar tissue on days 7, 14, and 21, respectively. Data are reported as mean and standard deviation (mean ± SD). ^*∗*,†,#^Statistically different between groups (*p* ≤ 0.05). ^*∗*^Compared to Sal and VH. ^*∗*†^Compared to Sal, VH, and SS. ^*∗*†#^Compared to Sal, VH, SS, and ES5.

The total number of cells and mast cells was higher in groups ES5 and ES10 on day 7 (F1) when compared to the control groups (Sal, VH, and SS). On day 14 (F2), group ES10 showed an increase on the amount of cells when compared to the other groups (Sal, VH, SS, and ES5), and on days 14 and 21, this same group showed a higher number of mast cells compared to groups Sal, VH, SS, and ES5 (Figure 3). Groups ES5 and ES10 have shown a higher vascularization in the wounds when compared to the control groups (Sal, VH, and SS) and on day 21 (F3) group ES10 showed an increase in the blood vessels number, when compared to the other groups (Figure 3).

**Figure 4:**
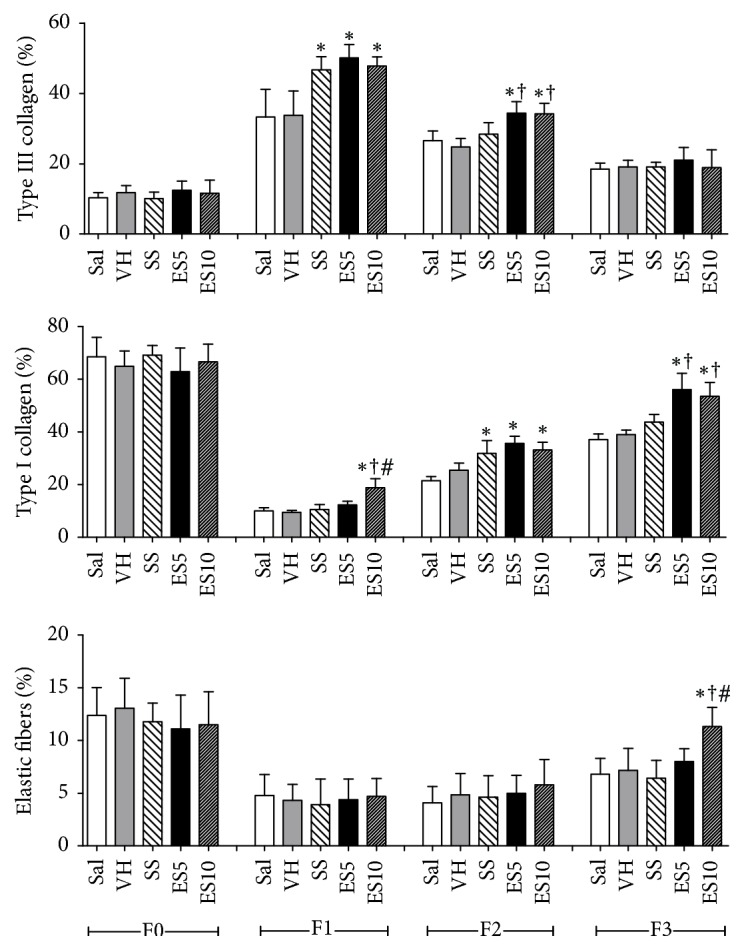
Proportion of type III collagen, proportion of type I collagen, and elastic fibers content in cutaneous wounds of diabetic rats treated with an ointment of* Strychnos pseudoquina* extract. Sal: 0.9% saline solution; VH: vehicle (lanolin cream); SS: silver sulfadiazine (cream 10 mg/g); ES5 and ES10:* S. pseudoquina* 5% and 10%, respectively. F0 = intact tissue on day 0; F1, F2, and F3 = scar tissue on days 7, 14, and 21, respectively. Data are reported as mean and standard deviation (mean ± SD). ^*∗*,†,#^Statistically different between groups (*p* ≤ 0.05). ^*∗*^Compared to Sal and VH. ^*∗*†^Compared to Sal, VH, and SS. ^*∗*†#^Compared to Sal, VH, SS, and ES5.

The number of type III collagen fibers on days 7 and 14 was higher in groups ES5 and ES10 when compared to Sal and VH groups. The number of type I collagen increased in group ES10 on days 7, 14, and 21 while in group ES5 this number increased on days 14 and 21. The elastic fibers content was greater in group ES10 on day 21 when compared to the other groups (Figure 4).

### 3.5. TGF-*β* Analysis

**Figure 5:**
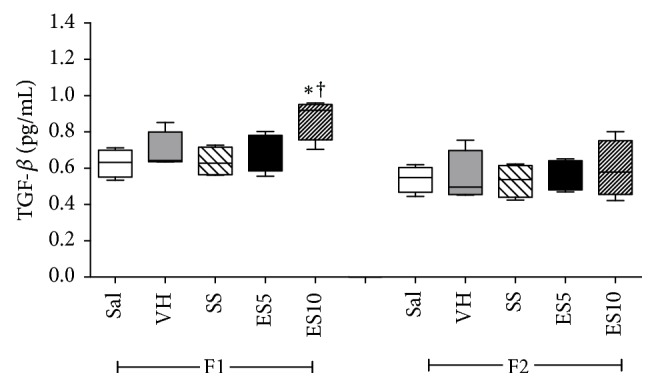
Levels of TGF-*β* in cutaneous wounds of diabetic rats treated with an ointment of* Strychnos pseudoquina* extract. Sal: 0.9% saline solution; VH: vehicle (lanolin cream); SS: silver sulfadiazine (cream 10 mg/g); ES5 and ES10:* S. pseudoquina* 5% and 10%, respectively. F1 and F2 = scar tissue on days 7 and 14, respectively. Data are reported as mean and standard deviation (mean ± SD). ^*∗*†^Statistically different compared to Sal, VH, and SS (*p* ≤ 0.05).

TGF-*β* levels increased on day 7 in group ES10 when compared to Sal, VH, and SS groups (Figure 5).

### 3.6. Oxidative Stress in the Healing Tissue

**Figure 6:**
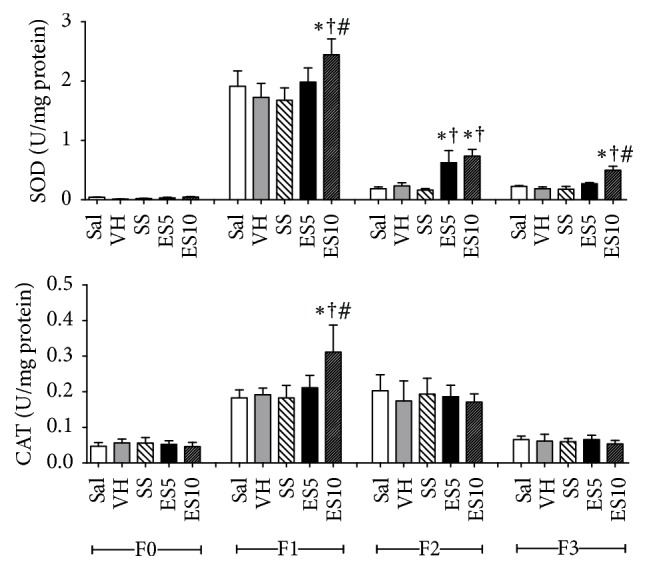
Levels of superoxide dismutase (SOD) and catalase (CAT) in cutaneous wounds of diabetic rats treated with an ointment of* Strychnos pseudoquina* extract. Sal: 0.9% saline solution; VH: vehicle (lanolin cream); SS: silver sulfadiazine (cream 10 mg/g); ES5 and ES10:* S. pseudoquina* 5% and 10%, respectively. F0 = intact tissue on day 0; F1, F2, and F3 = scar tissue on days 7, 14, and 21, respectively. Data are reported as mean and standard deviation (mean ± SD). ^*∗*,†,#^Statistically different between groups (*p* ≤ 0.05). ^*∗*†^Compared to Sal, VH, and SS. ^*∗*†#^Compared to Sal, VH, SS, and ES5.

SOD levels were higher in group ES10 on days 7 and 21 when compared to the other groups. On day 14, SOD levels were significantly higher in ES5 and ES10 groups when compared to Sal, VH, and SS groups. CAT levels also had higher values on day 7 in group ES10 when compared to the other groups (Figure 6).

**Figure 7:**
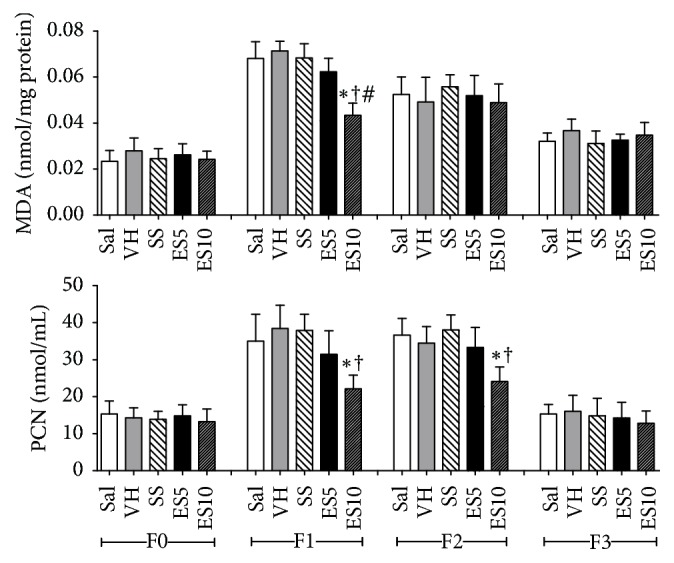
Levels of malondialdehyde (MDA) and carbonyl proteins (PCN) in cutaneous wounds of diabetic rats treated with an ointment of* Strychnos pseudoquina* extract. Sal: 0.9% saline solution; VH: vehicle (lanolin cream); SS: silver sulfadiazine cream (10 mg/g); ES5 and ES10:* S. pseudoquina* 5% and 10%, respectively. F0 = intact tissue on day 0; F1, F2, and F3 = scar tissue on days 7, 14, and 21, respectively. Data are reported as mean and standard deviation (mean ± SD). ^*∗*,†,#^Statistically different between groups (*p* ≤ 0.05). ^*∗*†#^Compared to Sal, VH, SS, and ES5.

MDA values were lower in ES10 group when compared to the other groups at day 7. Similar results were found for carbonyl proteins levels on days 7 and 14 (Figure 7).

## 4. Discussion

The polar extract of* S. pseudoquina* evaluated has mainly phenolic compounds. The high content of total polyphenols in the extract, with low flavonoid content, suggests the predominance of tannins in ES among phenolic compounds, since tannins were detected in the phytochemical prospecting by TLC. This fact can be explained by the use of the solvent ethyl acetate. Silva et al. [34] identified the presence of alkaloid indole, as nordiidrofluorcurarine, in addition to rutin and kaempferol 3-O-rutenoside in* S. pseudoquina* leaves extract. Another indole alkaloid, the nor-dihydrotoxiferine, has also been identified in bark extracts of this species [57]. Thus, the healing activity of stem bark of* S. pseudoquina* may be associated with the presence of these phenolic compounds, such as tannins and simple phenols, mainly due to the presence of chlorogenic acid. This metabolite has been described as present in other species of the* Strychnos* genus [58], but for the first time it is described for the* S. pseudoquina* species. It is already known in the literature that topical application of chlorogenic acid can accelerate the process of excision of wound healing by its ability to increase collagen synthesis as well as by its antioxidant property [59]. This evidence was reinforced by Bagdas et al. [60], which reported that chlorogenic acid intraperitoneally injected in streptozotocin-induced diabetic rats was effective in controlling the oxidative stress and accelerates skin wound healing in this model.

The toxicity of* Strychnos* species differs according to the part of the plant used and is mainly related to the presence of strychnine [32]. According to Santos et al. [61], the leaf extract from* S. pseudoquina* showed cytotoxicity in* Salmonella* strains TA98 in doses of 26.6 mg/plate. Other species, such as* Strychnos nux-vomica* and* Strychnos icaja*, have toxic activity due to the presence of strychnine and contain toxic alkaloids in their root bark [62].

Ointment of* Strychnos pseudoquina* extract in 5 and 10% concentrations was efficient to accelerate the wound closure and promotes the synthesis of matrix, enhancing the scar quality. To prove this hypothesis, silver sulfadiazine was used as a positive control, which is commonly used for wound treatment due its high antimicrobial and anti-inflammatory activity [44]. The results of this study showed that the* S. pseudoquina* treatment on diabetes had greater effect than silver sulfadiazine treatment because it accelerated the wound closure, promoting faster tissue reepithelialization and reducing the infection risk. Similar results were found in the topical treatment of wounds using essential oil from* Rosmarinus officinalis* in diabetic rats that presented higher wound contraction rate in 15 days of treatment [63].

Sarandy et al. [7] showed that* Brassica oleracea* leaves extract, a product rich in flavonoids and anthocyanins, is efficient in the topical treatment of cutaneous wounds with a complete wound closure in 20 days of treatment. Phytochemical analysis obtained from stem bark samples of* S. pseudoquina* showed phenolic compounds (i.e., flavonoids) and alkaloids, which are secondary metabolites described by its high therapeutic potential for diseases [63, 64]. Nicollete et al. [65] also found alkaloids and flavonoids in* S. pseudoquina* leaves. Aparecido Da Silva et al. [34] described the presence of these compounds in several* Strychnos* species. The authors found alkaloids as the main compound in this plant, while flavonoids and triterpenes were identified in reduced levels. The results obtained in this study confirm the great bioprospecting potential of* S. pseudoquina* extract, which is already used in Brazilian popular medicine to treat several diseases such as herpes [38], malaria [34], gastrointestinal disturbances [35], and gastric ulcer [36].

Animals that received ointment of* S. pseudoquina* extract, especially in the highest concentration, showed an increase in cellularity and vascularity, demonstrating high tissue metabolism during the repair in diabetes, mainly during the inflammatory phase [1]. Generally, in diabetes, the inflammatory phase is prolonged. There are defects of angiogenesis and cell proliferation and in formation and maturation of the granulation tissue resulting in a failed healing process [66]. Honório-França et al. [67] reported the hypoglycemic effect of among bark of* S. pseudoquina*. Romero-Cerecero et al. [68] demonstrated that the topical use of medicinal plants such as* Ageratina pichinchensis* in wound healing in diabetic rats stimulates an increase of cellularity and vascularity in the injured tissue. In addition to phagocytes and fibroblasts, mast cells are also found in abundance during the tissue repair process and play an important role in the production of angiogenic factors such as VEGF and TGF-*β*1, exerting a great influence on the proliferative response in the healing of cutaneous wounds [69]. In the present study, a dose-response increase in the mast cells number in groups treated with* S. pseudoquina* extract was observed. The increase in the number of these cells in diabetic rats was also observed in the topical use of ointment of* Schinus terebinthifolius* 5% extract, showing the important function of this cell in the inflammatory process [70]. The increase in cellularity and in mast cells number can justify the increase in TGF-*β* expression that was observed in this study [15]. TGF-*β* is considered to be a universal mediator that can be synthesized by different cells with positive action in the cutaneous repair process due its anti-inflammatory effect and capacity to decrease cell migration and accelerate the matrix synthesis [24, 71].

The remodeling phase is the last phase in the healing process which is characterized by an increase in the biomechanical resistance tissue due to the replacement of granulation tissue rich in type III collagen by the stronger tissue rich in type I collagen [45, 72]. The collagen and elastic fibers synthesis were higher in groups treated with* S. pseudoquina* probably due to the positive action of the extract compounds [34]. Gonçalves et al. [47] showed that the topical use of* Bathysa cuspidata* extract in cutaneous wounds promotes the synthesis of type I and type III collagen, accelerating the process of repair. The topical use of* Ageratina pichinchensis* extract also showed an enhancement of collagen fibers in the healing process in diabetic rats [68].

The balance between ROS production and the effect of the antioxidant system is extremely important for an efficient cutaneous repair [73]. When the tissue is damaged by the action of free radicals it is common to observe lipids, proteins, and cell DNA alterations, leading to an oxidative stress [13, 73]. The groups treated with* S. pseudoquina* showed a decline on the amount of oxidative stress markers (MDA and PCN) and an increase in the activity of antioxidant enzymes such as SOD and CAT. Together with our* in vitro* antioxidant assay, these results indicate that* S. pseudoquina* extract has a high antioxidant potential, which favors the cutaneous repair in diabetic rats. Topical treatment with* Albizia lebbeck* extract showed a significant phenols amount, with an increase of SOD as well as the reduction of MDA [74]. Studies using* Joannesia princeps* seed oil reduced the levels of carbonyl proteins in the wound area on day 14, helping the wound healing [75]. According to Zhang et al. [76],* Lens culinaris* extracts, which are rich in phenolic compounds, show a great antioxidant activity and therefore are efficient to maintain the tissue redox status. Studies with different medicinal plants have demonstrated the presence of isolated compounds such as tannic acids and chlorogenic acids, which showed significant antioxidant activities [77]. These findings show us that the compounds found in* S. pseudoquina* extract exhibited significant antioxidant activity and thus are a potent antioxidant therapeutic agent.

## 5. Conclusion

Our results indicate that the topical application of* S. pseudoquina* extract exerts a positive modulation on skin wound healing. When applied in preparations containing 5% and especially 10% of* S. pseudoquina* extract, the treatment stimulated a faster and efficient cutaneous repair in diabetic rats. The healing effects were partially related to the ability of the topical treatment in stimulating cellularity, TGF-*β* levels, collagen, and elastic fibers deposition and attenuate oxidative damage in scar tissue, accelerating wound closure. It is possible that the beneficial effect is related to the secondary metabolites identified in* S. pseudoquina* extract, especially tannins and simple phenols, including chlorogenic acid. Considering a biotechnological approach, further phytochemical studies of this extract are necessary to identify specific active components and elucidate its detailed mechanism of action.

## Figures and Tables

**Table 1:** Wound area (mm^2^) and wound contraction index (WCI; mm^2^/day) in diabetic rats treated with an ointment of *Strychnos pseudoquina* extract.

		Sal	VH	SS	ES5	ES10
F0	Area	160.3 ± 23.6	164.5 ± 13.6	165.3 ± 12.5	160 ± 12.1	166.5 ± 26.5
	WCI	00.0 ± 00.0	00.0 ± 00.0	00.0 ± 00.0	00.0 ± 00.0	00.0 ± 00.0
F1	Area	115.4 ± 15.9	113.1 ± 13.9	109.6 ± 12.1	95.5 ± 11.3^*∗*^	76.8 ± 16.06^*∗*^
	WCI	31.9 ± 17.4	39.7 ± 11.5	37.7 ± 13.1	32.4 ± 11.7	50.5 ± 12.6
F2	Area	30.0 ± 11.3	25.2 ± 12.9	35.3 ± 10.4	15.9 ± 8.8^*∗*^	16.8 ± 8.1^*∗*^
	WCI	84.7 ± 9.3	88.3 ± 7.9	81.3 ± 9.1	89.1 ± 6.05	89.9 ± 5.5
F3	Area	10.4 ± 3.4	9.1 ± 4.1	8.18 ± 3.6	2.8 ± 1.7^*∗*^	3.2 ± 1.5^*∗*^
	WCI	94.3 ± 5.3	94.0 ± 5.3	94.9 ± 2.6	98.0 ± 1.2	96.7 ± 2.4

Sal: 0.9% saline solution; VH: vehicle (lanolin cream); SS: silver sulfadiazine (cream) (10 mg/g); ES5 and ES10: *S. pseudoquina* 5% and 10%, respectively. F0 = intact tissue on day 0; F1, F2, and F3 = scar tissue on days 7, 14, and 21, respectively. Data are reported as mean and standard deviation (mean ± SD). ^*∗*^*p* ≤ 0.05, statistically different compared to Sal and VH.
